# Jarvis’s Adjuster

**Published:** 1850-09

**Authors:** 


JARVIS'S ADJUSTER.

An instrument, under this name, for adjusting fractures, and reducing dislocations has been before the profession for some years, but it has not received the attention its merits deserve. It enjoys the confidence, and has received the approbation of a number of eminent surgeons, among whom stands Professor Mott. We have had an opportunity of examining the instrument, and take pleasure in commending it as superior to anything of the kind we have seen, and we feel sure that a correct understanding of its merits, and the use of its powers, would do much towards stopping those suits for mal-practice, that are so annoying to Surgeons, and so injurious to the interests of the medical profession. It is time that some means were adopted for this object. A distinguished Professor in New York, Dr. James Webster, made "the frequency of suits of mal-practice," the subject of an introductory lecture, before the Geneva Medical College, last March, and no one can rise from the perusal of that lecture, without feeling that a better remedy is needed than the one suggested by Professor Webster. The abandonment of a particular class of patients, in order to avoid the risk of suits at law, is scarcely professional. The statistics of Professor Hamilton, and the quotation we made from Maclise, on the developements of broken bones in Dupuytren's Museum are not very creditable to modern surgery, and such things could not exist under the regulations of good surgery. We feel confident that if surgeons use the proper means, in the reunion of fractured bones, no justifiable claim for mal-practice would live long before a jury.

It is somewhat singular that in the Transactions of the American Medical Association, containing Professor Hamilton's deplorable statistics, on fractures, the admirable instrument of Dr. George O. Jarvis, of Connecticut, was condemned, by the committee on surgery. It is well known to our readers, that we prefer the bandage, in the treatment of fractures, to all other contrivances, but we feel no hesitation in saying, that, if we had not acquired, by long continued labors, the requisite art for applying the bandage, there is nothing that so nearly approaches that treatment, in its perfect adaptation to all the requirements of fractures, as Jarvis's adjuster. It has capacity for placing the skilful young surgeon on something like an equality with those who have gained much by experience.

In another number we shall discuss the merits of the excellent instrument of Dr. George O. Jarvis, and endeavor to show its usefulness to surgeons and physicians. The statistics of dislocations and fractures, display the limping gait of modern surgery, and we are solicitous to do all in our power to remedy the evil. B.

